# AZ32 Reverses ABCG2-Mediated Multidrug Resistance in Colorectal Cancer

**DOI:** 10.3389/fonc.2021.680663

**Published:** 2021-05-20

**Authors:** Kun Liu, Yan-Chi Li, Yu Chen, Xiao-Bao Shi, Zi-Hao Xing, Zheng-Jie He, Sheng-Te Wang, Wei-Jing Liu, Peng-Wei Zhang, Ze-Zhong Yu, Xue-Mei Mo, Mei-Wan Chen, Zhe-Sheng Chen, Zhi Shi

**Affiliations:** ^1^ Department of Cell Biology & Institute of Biomedicine, National Engineering Research Center of Genetic Medicine, MOE Key Laboratory of Tumor Molecular Biology, Guangdong Provincial Key Laboratory of Bioengineering Medicine, College of Life Science and Technology, Jinan University, Guangzhou, China; ^2^ Institute of Genomic Medicine, College of Pharmacy, Jinan University, Guangzhou, China; ^3^ State Key Laboratory of Quality Research in Chinese Medicine, Institute of Chinese Medical Sciences, University of Macau, Macau, China; ^4^ Department of Pharmaceutical Sciences, College of Pharmacy and Health Sciences, St. John’s University, Queens, NY, United States

**Keywords:** AZ32, ABCG2, multidrug resistance, colorectal cancer, CRISPR

## Abstract

Colorectal cancer is a common malignancy with the third highest incidence and second highest mortality rate among all cancers in the world. Chemotherapy resistance in colorectal cancer is an essential factor leading to the high mortality rate. The ATP-binding cassette (ABC) superfamily G member 2 (ABCG2) confers multidrug resistance (MDR) to a range of chemotherapeutic agents by decreasing their intracellular content. The development of novel ABCG2 inhibitors has emerged as a tractable strategy to circumvent drug resistance. In this study, an ABCG2-knockout colorectal cancer cell line was established to assist inhibitor screening. Additionally, we found that ataxia-telangiectasia mutated (ATM) kinase inhibitor AZ32 could sensitize ABCG2-overexpressing colorectal cancer cells to ABCG2 substrate chemotherapeutic drugs mitoxantrone and doxorubicin by retaining them inside cells. Western blot assay showed that AZ32 did not alter the expression of ABCG2. Moreover, molecule docking analysis predicted that AZ32 stably located in the transmembrane domain of ABCG2. In conclusion, our result demonstrated that AZ32 could potently reverse ABCG2-mediated MDR in colorectal cancer.

## Introduction

Multidrug resistance (MDR) is a frequent phenomenon that drastically limits the treatment of cancer patients. Accumulating evidence have demonstrated that the ATP-binding cassette (ABC) superfamily G member 2 (ABCG2), also known as breast cancer resistance protein (BCRP), confers resistance to a range of chemotherapeutic agents through extruding them to extracellular in an ATP dependent manner (1, 2). Human ABCG2 is predominantly located on the plasma membranes of cells in various tissues, such as small intestine, colorectal, gallbladder, testes, and capillary tissues, and it facilitates the function of blood–brain barrier, blood-testicular, and blood-placental barriers (3–5). Structurally, unlike its two functional homologs ABCB1 (P-glycoprotein) and ABCC1 (MRP1), ABCG2 is a half-transporter that only possesses one hydrophilic nucleotide binding domain in the N-terminal in the cytoplasm and one hydrophobic membrane-spanning domain containing six putative transmembrane helixes (6). Due to the unique structural architecture of ABCG2, the substrate profiles of it overlap with and yet differ from that of ABCB1 and ABCC1, consisting of topoisomerase inhibitors (i.e., mitoxantrone, SN38, topotecan, and doxorubicin), antimetabolites (i.e., 5-fluorouracil, and trimetrexatte), tyrosine kinase inhibitors (i.e., gefitinib, dasatinib, erlotinib, and sorafenib), photosensitizers (i.e., pheophorbide A and protoporphyrin IX), and fluorescent dyes (i.e., rhodamine 123 and Hoechst 33342) (7). Thus, ABCG2 significantly affects the absorption, distribution, metabolism, and efficacy of these compounds aforementioned. Potent inhibitors of ABCG2 have been identified in recent years, including fumitremorgin C (FTC) and its derivative ko143 (8, 9). Unfortunately, the side effect of FTC and ko143 restrain their development (10). We also reported several inhibitors of ABCG2 in decades (11–18). However, it is still necessary to identify novel inhibitors of ABCG2.

In this study, we found that ataxia-telangiectasia mutated (ATM) kinase inhibitor AZ32 was a potent inhibitor of ABCG2 and could sensitize ABCG2-overexpressing colorectal cancer cells to chemotherapeutic drugs mitoxantrone and doxorubicin by increasing their intracellular concentrations.

## Materials and Methods

### Reagents and Cell Culture

AZ32 (#T4443) was purchased from TargetMol (Shanghai, China). FTC (#118974-02-0) was obtained from BioBioPha (Kunming, China). Rhodamine 123 (#62669-70-9) was purchased from Sigma-Aldrich (Darmstadt, Germany). Mitoxantrone (#70476-82-3), doxorubicin (#25316-40-9), and cisplatin (#AA1A8019B) were purchased from D&B Biological Science and Technology (Shanghai, China), LC Laboratories (Massachusetts, USA) and Qilu Pharmaceutical (Jinan, China), respectively. 3-(4,5-dimethylthiazol-yl)-2,5-diphenyltetrazolium bromide (MTT) (#298-93-1) was purchased from Yuanye Bio-Technology (Shanghai, China). Polyetherimide (PEI) (#24765-1) was from Poly Sciences (Illinois, USA). Puromycin (#A1113803) was from Thermo Fisher Scientific (Shanghai, China). Anti-ABCG2 antibody (#sc-377176) was purchased from Santa Cruz Biotechnology (California, USA). Anti-β-tubulin antibody (#30302ES20) was purchased from YEASEN Biotech (Shanghai, China). HEK293T, human colorectal cancer cell line S1 and its drug-resistant cell line S1-M1-80 with ABCG2 overexpression (19) were cultured in DMEM containing 10% bovine serum at 37°C in a humidified atmosphere of 5% CO_2_.

### Vector Generation and Lentivirus Infection

LentiCRISPRv2 vector (from Addgene #52961) was digested with BsmB I and liagted with annealed oligonucleotides (ABCG2-SgRNA-F: 5’-CACCGGCTGCAAGGAAAGATCCAAG-3’, ABCG2-SgRNA-R: 5’-AAACCTTGGATCTTTCCTTGCAGCC-3’). HEK293T cells were transfected using PEI at 70% confluency with recombinant vectors and packaging vectors pMD2G and psPAX2. The viral supernatant was harvested after 72 h of transfection. S1-M1-80 cells were infected with viral supernatant containing 10 µg/ml polybrene, and were selected with 30 µg/ml puromycin to establish the stable cell lines. Finally, a monoclonal S1-M1-80 cell line with stable knockout of ABCG2 was acquired by single-cell culture.

### Western Blot Assay

Cells were trypsinized and washed twice with cold PBS, then resuspended and lysed in RIPA buffer (1% NP-40, 0.5% sodium deoxycholate, 0.1% SDS, 10 ng/ml PMSF, 0.03% aprotinin, 1 µM sodium orthovanadate) at 4°C for 30 min. Lysates were centrifuged for 10 min at 14,000×*g* and supernatants were stored at −80°C as whole cell extracts. Proteins were separated on 10% SDS-PAGE gels and transferred to polyvinylidene difluoride membranes. Membranes were blocked with 5% BSA and incubated with the indicated primary antibodies. Corresponding horseradish peroxidase-conjugated secondary antibodies were used against each primary antibody. Signals were detected with the ChemiDoc XRS chemiluminescent gel imaging system (Analytik Jena).

### Genomic PCR and Sequencing Analysis

The genomic DNA of cells was extracted with the QuickExtractDNA extraction kit following the manufacturer’s protocol and amplified with primer (ABCG2-F: 5’-GAGATATATAGCATGTGTTGGAGGG-3’, ABCG2-R: 5’-CTATCAGCCAAAGCACTTACCC-3’) designed for the target region of interest using a Pfu DNA polymerase. The PCR product was sequenced after agarose gel electrophoresis.

### Cytotoxicity Assay

Cells were seeded into a 96-well plate at a density of 8,000 cells/well. Chemotherapeutic agents with different concentrations were added after preincubated in the presence or absence of inhibitors for 1 h. After 68 h of incubation, MTT (500 μg/ml) was added to each well. The solution in the wells was discarded, and the dark-blue formazan crystals were dissolved in 50 μl DMSO. Absorbance was measured at 570 nm by a microplate reader (Bio Tek Instrument).

### Drug Accumulation Assay

Cells in 6-well plate with a concentration of 3.5 × 10^5^ cells/well preincubated with or without inhibitors for 1 h, then mitoxantrone, doxorubicin and rhodamine 123 were added with 10 μM for another 2 h, respectively. After washed three times with PBS, these compounds accumulated in the cell were observed and quantified by fluorescence microscope (Olympus) and flow cytometer (Beckman), respectively.

### Docking Analysis

The Crystal structure of ABCG2 was obtained from Protein Data Bank (PDB), and the 3D structures of small molecules, including AZ32, FTC, and doxorubicin, were downloaded from PubChem. All docking calculations were performed using AutoDock Vina, and the results were visualized by PyMOL (20).

### Statistical Analysis

All experiments were performed at least three times, and differences among each group were determined by one-way ANOVA. P-value <0.05 was considered as statistical significance.

## Results

### Establishment ABCG2-Knockout Colorectal Cancer Cells

**Figure 1:**
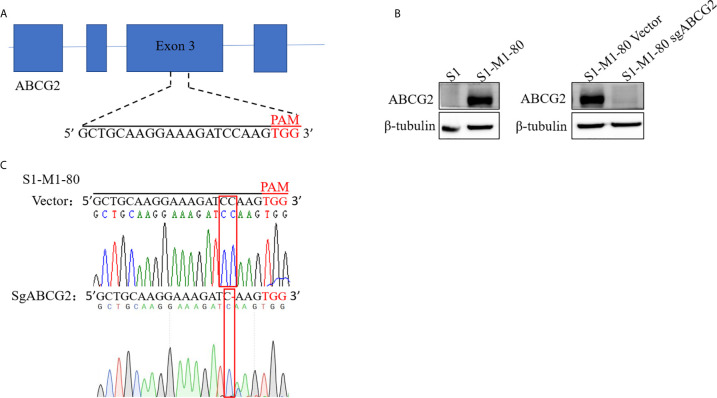
Establishment ABCG2-knockout colorectal cancer cells. A schematic diagram of the designed sgRNA targeting ABCG2 in exon 3 is shown **(A)**. The protein expression levels of ABCG2 were examined by Western blot, and β-tubulin was used as loading control **(B)**. The sequencing comparison and original data of S1-M1-80 cells are shown **(C)**.

To establish ABCG2 knockout cell line with CRISPR-Cas9 system, we firstly generated lentiCRISPRv2 vector which contains a targeting sequences from exon 3 of human ABCG2 gene end with a 5’NGG3’ protospacer adjacent motif (PAM) sequence **(** **Figure 1A** **)**. S1-M1-80 cells were selected with puromycin after transduction with LentiCRISPRv2 viral supernatant. A monoclonal S1-M1-80 cell line with stable knockout of ABCG2 was acquired by single-cell culture, and its protein levels of ABCG2 were undetectable by western blot **(** **Figure 1B** **)**. The further sequencing results of genomic DNA PCR productions showed that a “C” base was deleted in the target position of S1-M1-80 sgABCG2 cells in comparison to S1-M1-80 Vector cells **(** **Figure 1C** **)**. These results indicate that ABCG2-knockout colorectal cancer cells were successfully established.

### AZ32 Sensitizes ABCG2-Overexpressing Colorectal Cancer Cells to ABCG2-Substrate Chemotherapeutic Drugs

**Figure 2:**
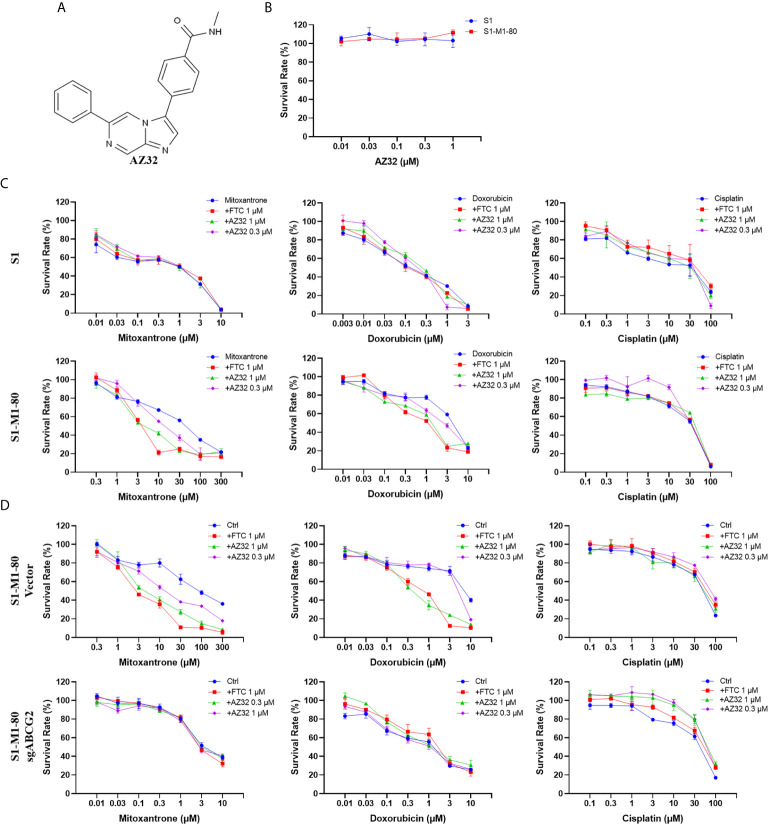
AZ32 sensitized ABCG2-overexpressing colorectal cancer cells to ABCG2-substrate chemotherapeutic drugs. The chemical structure of AZ32 is shown **(A)**. Cells were treated with the indicated concentrations of AZ32 or other agents for 72 h, and cell survival was measured by MTT assay. The representative growth curve of cells treated with AZ32 alone **(B)** or in combination with mitoxantrone, doxorubicin and cisplatin **(C, D)** are shown.

AZ32 is a novel ATM inhibitor (21), and its chemical structure is shown in **Figure 2A**. To investigate the effect of AZ32 on ABCG2-mediated MDR in colorectal cancer cells, we firstly examined the cytotoxicity of AZ32 in the ABCG2-overexpressing MDR colorectal cancer cells S1-M1-80 and its parental S1 cells. The results showed that AZ32 at the used concentrations were non-cytotoxic in both S1 and S1-M1-80 cells **(** **Figure 2B** **)**. We then detected the cytotoxicity of combination of AZ32 with two ABCG2 substrates, mitoxantrone and doxorubicin, and one non-ABCG2 substrate, cisplatin, at the various concentrations. As shown in **Figures 2C, D**, S1-M1-80 and S1-M1-80 Vector cells showed much higher resistance to mitoxantrone and doxorubicin but not cisplatin than S1 and S1-M1-80 sgABCG2 cells, respectively. Compared with the well-known ABCG2 inhibitor FTC, AZ32 showed mildly weaker effect on reversing the resistance of S1-M1-80 and S1-M1-80 Vector cells to mitoxantrone and doxorubicin but not cisplatin. Neither AZ32 nor FTC increased the cytotoxicity of the above chemotherapeutic drugs in S1 and S1-M1-80 sgABCG2 cells. These data suggest that AZ32 can sensitize ABCG2-overexpressing colorectal cancer cells to ABCG2-substrate chemotherapeutic agents.

### AZ32 Enhances the Intracellular Accumulation of ABCG2 Substrates in ABCG2-Overexpressing Colorectal Cancer Cells

**Figure 3:**
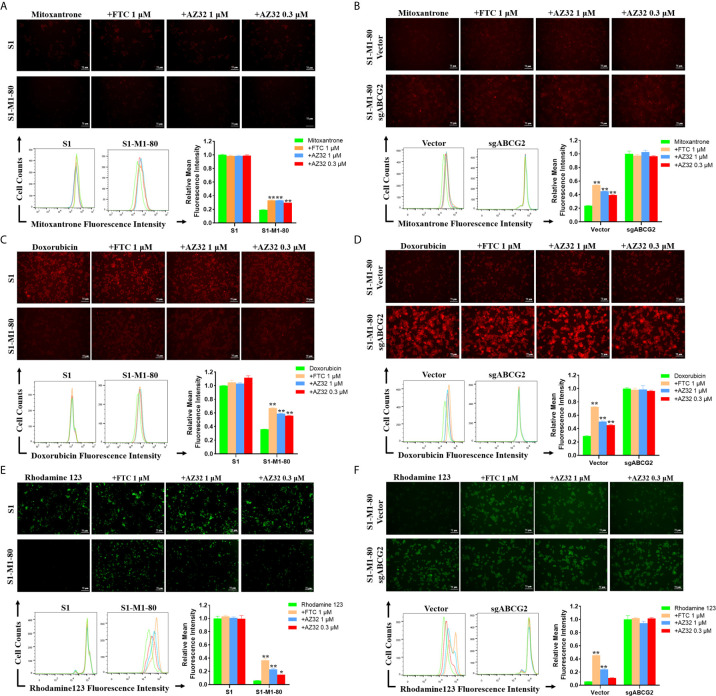
AZ32 enhances the intracellular accumulation of ABCG2 substrates in ABCG2-overexpressing colorectal cancer cells. Cells were incubated with 10 μM mitoxantrone, doxorubicin or rhodamine 123 for another 2 h at 37°C after pre-treated with the indicated concentrations of AZ32 or FTC for 1 h at 37°C and photographed by fluorescent microscope. Then the florescent intensity was measured by flow cytometer and quantified **(A–F)**. ^*^ *P <*0.05, and ^**^ *P <*0.01 vs. corresponding group.

To examine whether AZ32 reversed ABCG2-mediated MDR in colorectal cancer cells is due to inhibition of the transporter activity of ABCG2, we detected the intracellular levels of three ABCG2 substrates mitoxantrone, doxorubicin and rhodamine 123 in the presence or absence of AZ32. As shown in **Figures 3A–F**, S1-M1-80 and S1-M1-80 Vector cells showed much weaker intracellular levels of mitoxantrone, doxorubicin and rhodamine 123 than S1 and S1-M1-80 sgABCG2 cells, respectively. Compared with FTC, AZ32 showed mildly weaker effect on enhancing the intracellular levels of mitoxantrone, doxorubicin and rhodamine 123 in S1-M1-80 and S1-M1-80 Vector cells. Neither AZ32 nor FTC enhanced the intracellular levels of mitoxantrone, doxorubicin and rhodamine 123 in S1 and S1-M1-80 sgABCG2 cells. These results suggest that AZ32 can enhance the intracellular accumulation of ABCG2 substrates by inhibiting the transporter activity of ABCG2 in colorectal cancer cells.

### AZ32 Does Not Alert the Protein Expression of ABCG2 in Colorectal Cancer Cells

**Figure 4:**
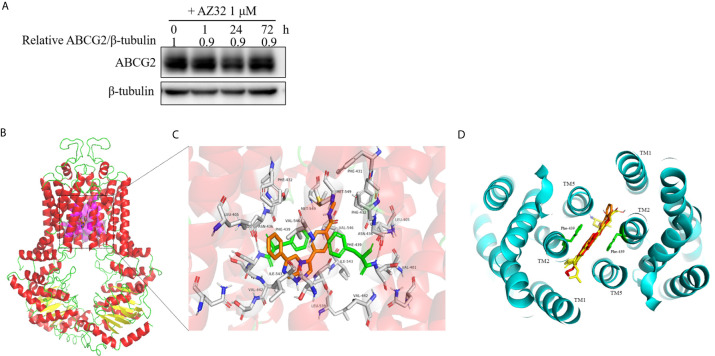
AZ32 does not alter the protein expression of ABCG2 in colorectal cancer cells and model for binding of AZ32 to ABCG2. S1-M1-80 cells were treated with AZ32 at 1 μM for the indicated time points. The protein expression levels of ABCG2 were examined by Western blot, and β-tubulin was used as loading control **(A)**. The optimal docked pose of AZ32 within the putative multidrug-binding site of human ABCG2 based on the crystal structure available from PDB. ABCG2 conformation is presented as a ribbon diagram and colored by secondary structure: read-helix, yellow-sheet, green-loop. AZ32 is shown as stick mode within a slit-like cavity of ABCG2, and the binding surface is exhibited as magenta **(B)**. Zoomed-in the highlighted area shows that AZ32 is sandwiched between Phe-439 (colored green) side chains, and AZ32 interacts favorably with the hydrophobic residues Val-401, Leu-405, Phe-431, Phe-432, Leu-439, Val-442, and Ile-543 (shown as sticks, labeled) **(C)**. The binding site of AZ32 (orange sticks) overlaps with FTC, mitoxantrone and doxorubicin (displayed as yellow, green and red sticks respectively), viewed from the extracellular space of the structure of ABCG2 **(D)**.

The reversal of ABCG2-mediated MDR can be accomplished by either inhibiting its transporter activity or downregulating its expression. To examine the effect of AZ32 on the protein expression of ABCG2, S1-M1-80 cells were treated with AZ32 at various periods. As shown in **Figure 4A**, AZ32 does not alert the protein expression of ABCG2 for up to 72 hours.

### Model for Binging of AZ32 to ABCG2

A slit-like cavity close to the two-fold symmetry of ABCG2 dimerization was acknowledged as the ligands binding pocket of ABCG2 (22). Therefore, a structure-based docking assay was conducted to validate the binding of AZ32 with ABCG2. The predicted binding mode showed that AZ32 was located in the crevice between ABCG2 monomers (**Figure 4B**), and it stabilized in this slit-like cavity mainly through hydrophobic contact with other hydrophobic residues on the binding surface. In this conformation (**Figure 4C**), AZ32 was located in the transmembrane domain of ABCG2 surrounded by multiple hydrophobic amino acids, including Leu-405, Phe-431, Phe-432, Val-442, and Ile-543. In addition, the aromatic rings of AZ32 were sandwiched between the phenyl moiety of Phe-439 from opposing monomers *via* π–π stacking. Furthermore, Met-549 on TM5 of ABCG2 interplayed with the benzene ring of AZ32 through π-sulfur interaction. Furthermore, AZ32 almost completely overlayed with FTC, mitoxantrone and doxorubicin in the putative drug-binding cavity of ABCG2 **(** **Figure 4D** **)**, suggesting that AZ32 may inhibit the transporter activity of ABCG2 by competing with the substrates to bind ABCG2.

## Discussion

Despite ample advances in novel cytotoxic and targeted agents, resistance to chemotherapeutic drugs continues to be one of the biggest obstructions in the treatment of patients with metastatic colorectal cancer (23–25). With accumulated evidence, transmembrane transporter ABCG2 has emerged as an attractive targeting moiety to combat chemotherapeutic drugs resistance (26, 27). Abcg2^−/−^ knockout and wild-type mice were widely used to study the effect of ABCG2 on the tissue distribution of potential substrates by analyzed their plasma, small intestine, colorectal, liver, kidneys, and testicles (28, 29). Recently, Daniella et al. established ABCG2-knockout and EGFP tagged ABCG2 reporter cell lines in human lung adenocarcinoma cells, which were useful to study the ABCG2 gene regulation and visualizing protein activity in live cells (30). In this study, we established an ABCG2-knockout human colorectal cancer cell line by CRISPR-Cas9 mediated genome editing technology which we have used previously (31, 32). This precision-engineered colorectal cell line provided a valuable model for screening new ABCG2 inhibitors and validating the specificity of potential inhibitors.

As a novel selective inhibitor of ATM kinase, AZ32 significantly potentiated the radiotherapy effect on glioma *in vitro* and *in vivo* (21). In the present study, we found that AZ32 could sensitize ABCG2-overexpressing colorectal cancer cells to mitoxantrone and doxorubicin but not cisplatin. Further results showed that AZ32 could enhance the intracellular accumulation of mitoxantrone, doxorubicin, and rhodamine 123 in ABCG2-overexpressing colorectal cancer cells. Western blot assay indicated that AZ32 did not alter the expression of ABCG2. Moreover, the predicted molecule docking model presented that AZ32 was stably located in the transmembrane domain of ABCG2. All these data suggest that AZ32 could inhibit the transporter activity of ABCG2 to reverse ABCG2-mediated multidrug resistance in colorectal cancer by competing with the substrate chemotherapeutic drugs to bind ABCG2. However, the combined effect of AZ32 with ABCG2-substrate chemotherapeutic drugs in colorectal cancer need to be further validated *in vivo*.

In conclusion, our result demonstrated that AZ32 could potently reverse ABCG2-mediated MDR in colorectal cancer by inhibit the transporter activity of ABCG2, which is supported by the predicted binding mode that presented the hydrophobic interactions of AZ32 within the drug binding cavity of ABCG2. Therefore, the combination of AZ32 with ABCG2-substrate chemotherapeutic drugs may be a potential strategy to overcome MDR in colorectal cancer.

## Data Availability Statement

The original contributions presented in the study are included in the article/**Supplementary Material**. Further inquiries can be directed to the corresponding authors.

## Author Contributions

KL, Y-CL, YC, M-WC, Z-SC, and ZS designed the experiments, performed the experiments, analyzed the data, and wrote the paper. X-BS, Z-HX, Z-JH, S-TW, W-JL, P-WZ, Z-ZY, and X-MM performed the experiments and wrote the paper. All authors contributed to the article and approved the submitted version.

## Funding

This work was supported by funds from the National Key Research and Development Program of China No. 2017YFA0505104 (ZS), the National Natural Science Foundation of China Nos. 81772540 (ZS) and 51922111 (M-WC), the Science and Technology Program of Guangdong No. 2019A050510023 (ZS) and the Science and Technology Development Fund, Macau SAR No. 0004/2019/AGJ (M-WC).

## Conflict of Interest

The authors declare that the research was conducted in the absence of any commercial or financial relationships that could be construed as a potential conflict of interest.
