# Supplementary figures and images for: AZ32 Reverses ABCG2-Mediated Multidrug Resistance in Colorectal Cancer
Source: Front Oncol. 2021 May 20;11:680663. doi: 10.3389/fonc.2021.680663 (PMC8173085; doi:10.3389/fonc.2021.680663)

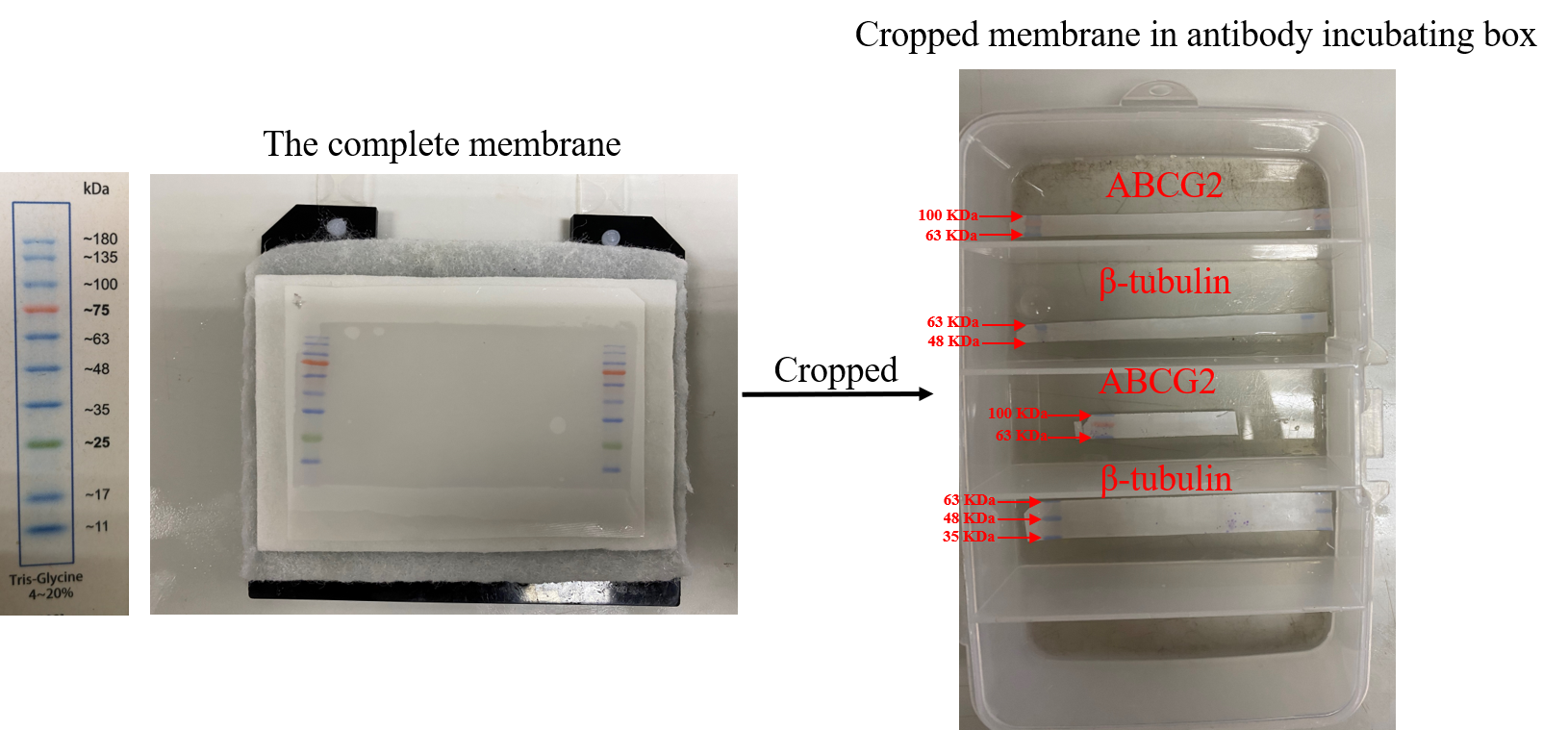

Supplement: Supplementary file 1 [file Image_1.tif]
